# Measuring compassionate healthcare with the 12-item Schwartz Center Compassionate Care Scale

**DOI:** 10.1371/journal.pone.0220911

**Published:** 2019-09-05

**Authors:** Ana Maria Rodriguez, Beth A. Lown

**Affiliations:** 1 PatientsLikeMe, Boston, Massachusetts, United States of America; 2 School of Physical and Occupational Therapy, McGill University, Montreal, Canada; 3 The Schwartz Center for Compassionate Healthcare, Boston, Massachusetts, United States of America; 4 Harvard Medical School, Department of Medicine, Boston, Massachusetts, United States of America; Florida International University, UNITED STATES

## Abstract

**Background:**

Patients and clinicians endorse the importance of compassionate healthcare but patients report gaps between its perceived importance and its demonstration. Empathy and compassion have been associated with quality of life and significant health outcomes but these characteristics are not optimally measured or used for performance and organizational improvement.

**Objective:**

To address these gaps, we conducted a study with the objective of evaluating the properties of the 12-item Schwartz Center Compassionate Care Scale^®^ using psychometric analysis and cognitive debriefing.

**Methods:**

Non-hospitalized patients with multiple chronic conditions were sampled using an on-line platform. Classical test theory and Rasch measurement theory were used to evaluate psychometric properties of the scale. Structured questions elicited cognitive responses regarding clarity of each item.

**Results:**

Classical test theory analysis confirmed that the 12-item Schwartz Center Compassionate Care Scale is a unidimensional scale with excellent internal consistency and test-retest reliability. Patients’ ratings of compassionate behaviors using the Schwartz Center Compassionate Care Scale correlated significantly with a related instrument designed to measure empathy, demonstrating convergent validity. Rasch measurement theory showed that reducing the number of response options on 3 items in the scale would improve respondents’ discrimination between responses on these items. Although person-item threshold distribution analysis showed that patients may wish to rate compassionate care at levels both higher and lower than the scale permits, items could be ordered on an interval scale from low to high levels of compassionate care.

**Conclusions:**

The current 12-item Schwartz Center Compassionate Care Scale demonstrates excellent psychometric properties by Classical Test Theory and Rasch measurement theory. The 12-item Schwartz Center Compassionate Care Scale adds questions related to understanding and discussing emotional, contextual issues and the needs of the patient and family. Easily completed on-line, it could be used for work-place based assessment and feedback to clinicians and performance or quality improvement.

## Introduction

Compassion for those who suffer is fundamental to the purpose of healthcare. Compassion represents a family of other-oriented emotions that includes empathic concern, caring, and tenderness for one who is suffering [1]. Extensive research has shown that these emotions, elicited by and congruent with the perceived welfare of someone in need, evokes a motivational state to ameliorate his or her concerns, pain, distress and suffering [1], [2]. Compassion is often conflated with the concepts of empathy and sympathy. Patients may perceive empathy as efforts to understand their situation and perspective, whereas sympathy may be perceived as pity [3]. Compassion enacted by healthcare providers adds the action component of helping behaviors.

Compassionate healthcare is characterized by relationships based on empathy, concern and respect for persons, contextualized knowledge of the patient as an individual within a network of relationships at home and in his or her communities, effective communication within interactions, over time, and across settings, and facilitation of patients’ and families’ participation in decisions and care [4]. Patients’ and families’ perceptions of providers’ empathy and compassion have been correlated with patient trust, satisfaction and health enablement [5], as well as clinical outcomes such as shortened duration of viral illness [6], improved treatment outcomes, including quality of life after hospitalization for a traumatic injury [7], and improved psychosocial adjustment and quality of life among cancer patients [8]. Physicians’ self-reported compassion and empathy have been correlated with improved control of chronic diseases such as diabetes and decreased hospitalizations for their serious complications [9] [10]. A meta-analysis of studies of interventions to improve patient-clinician relationships showed significant effects on health outcomes for patients with various chronic conditions [11]. Despite the importance ascribed to compassionate healthcare by professionals, patients, and their family members, prominent examples of the lack of such care have raised concerns internationally [12].

Some measures have been developed to measure compassion. These include competence among nurses (Compassion Competence Scale), patient reports of the compassion in the care received (the Compassion Scale, the Compassionate Care Assessment Tool), and the Compassion Practices Scale, a measure of organizational support for compassionate care [13]. However, patients’ perceptions of compassion are not routinely measured in actual practice. This makes it challenging to identify lapses or improve performance by providing feedback to healthcare professionals to enhance the expression of their compassion towards patients and their family members. The aim of this study was to assess the psychometric characteristics of the 12-item Schwartz Center Compassionate Care Scale in preparation for an exploration of its utility and application to quality and performance improvement, and for educational purposes.

## Development of the Schwartz Center Compassionate Care Scale

The Schwartz Center for Compassionate Healthcare, a nonprofit organization dedicated to promoting compassion and supporting care providers in healthcare organizations, developed criteria to assess the compassionate care provided by professional caregivers [14]. The initial criteria were developed by a 20-member working group composed of individuals with diverse viewpoints and experience, including cancer survivors, individuals suffering from chronic pain and/or debilitating illnesses, family members of patients, interdisciplinary healthcare providers and individuals working in healthcare policy and advocacy. Based on this work, the Schwartz Center developed a set of 16-items to assess compassionate care in recently hospitalized patients [4]. These 16 items were tested with recently hospitalized patients in the U.S [14] and subsequently in Ireland with recently hospitalized and non-hospitalized patients [15]. Patients’ ratings of the importance of the elements of compassionate care were similar in the U.S. and Ireland. Importantly, both studies revealed gaps between patients’ ratings of the importance of specific behaviors and their successful demonstration by professional staff during a recent hospitalization. A first psychometric assessment of the measure was completed and items with the best factor loading were selected to generate the unidimensional 12-item scale, that was called the Schwartz Center Compassionate Care Scale (SCCCS) [14].

The psychometric properties of the 12-item scale have not yet been evaluated. The primary objective of this study was to review, to psychometrically evaluate, and to potentially refine the 12-item version of the SCCCS. Specific objectives were to 1) review the content validity of the items of the SCCCS; 2) to estimate the other psychometric properties of the SCCCS using Classical Test and Modern Test Theory Methods (Rasch Measurement Theory).

## Study methods

### Ethics statement

Ethics Approval and Consent to Participate: All procedures performed in studies involving human participants were in accordance with the ethical standards of the institutional and/or national research committee and with the 1964 Helsinki declaration and its later amendments or comparable ethical standards. Ethical review of study procedures and approval were obtained from both the Massachusetts General Hospital and the New England Institutional Review Board. Informed consent was obtained from all individual participants included in the study.

The authors confirm that all patient/personal identifiers have been removed or disguised so the patient/person(s) described are not identifiable and cannot be identified through the details of the story.

### Participants

Patients were recruited from an online patient community (PatientsLikeMe, Inc., hereafter referred to as PLM). PLM has hundreds of thousands of registered patient-members representing more than 2,500 conditions. Patients joining the site are asked to share information about their diseases through customized questionnaires that populate their profile. Patients may also agree to be contacted and potentially invited to participate in research studies.

To participate in this study, PLM patient-members had to be adult males and females residing in the United States with at least one of the following primary chronic conditions: cancers (all), neurologic disorders (Amyotrophic Lateral Sclerosis, multiple sclerosis, Parkinson’s disease), heart disorders (coronary artery disease, hypertension, congestive heart failure, cardiomyopathy), and lung and respiratory disorders (pulmonary fibrosis, asthma, chronic obstructive pulmonary disease, cystic fibrosis, emphysema, pulmonary hypertension), or a mental illness. They additionally had to have been hospitalized within the last 18 months to ensure that they could evaluate compassion in the acute care setting. Non-English speaking patients were excluded from the study.

### Survey procedures and phases

The psychometric validation study consisted of two parts: 1) content validity, using syntactic and qualitative evaluation of the SCCCS; 2) quantitative psychometric evaluation of the 12-item version of the SCCCS. Between December 2015 and February 2016, 99 eligible patients were sent emails inviting them to participate in the qualitative part of the research study, of which 23 completed the cognitive debriefing survey (referred to as the cognitive debriefing sample). 682 eligible patients were sent emails inviting them to participate to the quantitative psychometric assessment part of the study, of which 167 completed the quantitative psychometric assessment survey (referred to as the psychometric sample). More than half of these participants were invited to fill the questionnaires a second time, approximatively 2 weeks after their first completion. It was completed by 61 patients.

Sociodemographic information was collected on participating patients including gender, age, main chronic condition, ethnicity, household income, and the highest level of education completed. Participating patients were asked to complete the 12-item SCCCS (S1 Table) and the Consultation and Relational Empathy (CARE) Measure [16]. The methodology and results for the content (face) validity of the measure by syntactic and qualitative evaluation of the SCCCS are presented in S2 and S3 Tables.

Ethical review of study procedures and approval were obtained from both the Massachusetts General Hospital and the New England Institutional Review Boards. Patients who indicated having one of the included primary conditions were invited by email to participate in the study. Patients were given the opportunity to ask questions about the study. If patients clicked on the link provided, they agreed to participate in the research study.

### Measures

#### Schwartz Center Compassionate Care Scale (SCCCS)

The 12-item SCCCS is the revision of the 16-item version of the same measure. The psychometric properties of the 16-item SCCCS indicated strong reliability when used to rate individual physicians (Cronbach’s alpha = 0.97 and 0.95). A one-factor model was a good fit for all items, and the unidimensionality of the scale was supported using Mokken analysis. During the psychometric evaluation, 4 items were identified as problematic as it did not fit the factor solution and suggested to be removed from the measure [14].

#### Consultation and Relational Empathy Scale (CARE)

The CARE Measure is a patient-reported questionnaire that measures empathy in the context of healthcare interactions during a consultation between a clinician and a patient, primarily in primary care and outpatient settings [16]. The CARE Measure consists of 10 questions associated with 5-points Likert scales ranging from “Poor” to “Excellent.” The CARE Measure has some items that are similar to the SCCCS; for example, *Showing care and compassion*, and *Listening and explaining things clearly*. Response options are rated from 1 to 5 and summed for a total score, ranging from 10 to 50. The psychometric properties of the CARE measure have been shown to have face and content validity, high concurrent validity (*r* = 0.84, *p* < 0.001 with the Reynolds Empathy Measure (RES) and *r* = 0.77, *p* < 0.001 with the Barrett-Lennard empathy subscale (BLESS) and high internal reliability (Cronbach’s alpha = 0.92) in general practice [16] and in specialty care [17], [18].

## Statistical methods

Descriptive statistics were used to characterize the participants: mean values and standard deviations for continuous variables, and frequencies and percentages for categorical variables. Floor and ceiling effects were calculated per item.

Classical Test Theory (CTT) was initially used to assess the psychometric properties of the SCCCS. CTT assumes that the measure of a person’s score is the sum of its true score and a random error [19]. The goal of CTT is to estimate the reliability of the score, or the importance of the random error respective to the total score. Content validity was assessed with the qualitative assessment of the SCCCS (S2 and S3 Tables). Internal consistency reliability was assessed by item-to-total correlation and Cronbach’s alpha coefficient [20], [21]. Test-retest reliability was assessed by comparing the consistency of scoring of the SCCCS at the two administration times using Intraclass Correlation Coefficients (ICC 1,2).

Convergent validity (construct validity) was assessed using the Pearson correlation coefficient between the SCCCS scale and the CARE. It was hypothesized that the correlation between the SCCCS and the CARE would be positive and moderate (between 0.40 and 0.59).

To estimate factorial validity, an exploratory factor analysis (EFA) was performed. The numbers of optimal factors were determined using both rotated and oblique factor solution. The factor structure determined by the EFA was further confirmed using Confirmatory Factor Analysis (CFA) and appropriate fit statistics (X^2^ prob, Root Mean Square Error of Approximation, Root Mean Square Residual, Goodness of fit Index, Bentler Comparative Fit Index).

To re-evaluate the scaling properties and construct validity of the SCCCS, Rasch measurement theory (RMT) was used. RMT analysis is a probabilistic modelling technique used to assess whether data accord with model expectations and whether the internal construct validity of the scale is supported [22][23]. RMT analyses used to assess whether the SCCCS conformed to RMT model expectations are briefly presented and are explained in more detail elsewhere [24], [25]:

1) Fit to the RMT model was assessed using fit residual mean values and summary and individual item chi-square statistics;

2) Internal reliability was assessed using the Person Separation Index (PSI), or the extent to which items can distinguish between distinct levels of compassionate care (analogous to Cronbach’s alpha when the distribution is normal);

3) Item category thresholds determined whether response categories were understood by patients;

4) The unidimensionality of the SCCCS or the confirmation that the SCCCS is measuring a single dimension was assessed using independent t-tests;

5) The magnitude of residuals was assessed by verifying response dependency, when two items’ answers are correlated and overestimating the reliability estimates;

6) The item difficulty and person ability ranges on the same log-odd units scale helped to establish targeting, or the match of patients’ ratings of compassionate care and the level of compassionate care measured by the items and response options;

7) Differential item functioning (DIF), was assessed for gender, age group, household income group, ethnic group, and time since admission, using analysis of variance (ANOVA, alpha = 0.05).

A partial credit Rasch polytomous model was used. SAS 9.4 and RUMM2030 were used to complete the statistical analyses.

## Results

### Participant characteristics

As seen in Table 1, for the quantitative assessments, most patients were female (74.7% for the psychometric sample but more males (65.2%) were part of the cognitive debriefing sample. Patients were on average 57 and 56 years old (range from 23 to 84 overall). The majority of both groups had some college education (52.2% and 47.9%, respectively).

**Table 1:** Sociodemographic characteristics: Cognitive debriefing (qualitative) and psychometric (quantitative) phases.

*Characteristics*	Cognitive Debriefing Sample Mean (SD) or Frequency (%) Total n = 23	Psychometric Sample Mean (SD) or Frequency (%) Total n = 167
Age	57.7 (13.1) (Range: 33–75)	56.3 (11.1) (Range: 23–84)
Age Group		
18 to 39 years	3 (13.0)	10 (6)
40 to 55 years	5 (21.7)	68 (40.7)
56 to 65 years	7 (30.4)	54 (32.3)
Above 65 years	8 (34.8)	35 (21.0)
Gender (missing n = 1)		
Female	8 (34.8)	123 (74.7)
Male	15 (65.2)	43 (25.7)
Non-Hispanic	22 (95.7)	160 (95.8)
Education (missing n = 4)		
Some high school or High School	3 (13.0)	18 (10.8)
Some College	12 (52.2)	80 (47.9)
College	4 (17.4)	41 (24.6)
Graduate Education	4 (17.4)	24 (14.4)
Household Income (missing n = 26)		
0-25K$	7 (30.4)	39 (23.4)
36-50K$	5 (21.7)	33 (19.8)
50-100K$	3 (13.0)	42 (25.2)
100-200K$	2 (8.7)	22 (13.2)
200K$ and above	1 (4.4)	5 (3.0)
Time Since Last Hospital Admission (missing n = 2)		
Within the last 90 days	2 (8.7)	47 (28.1)
Within the last 6 months	1 (4.4)	53 (31.7)
Within the last 12 months	14 (60.9)	65 (38.9)

**Fig 1:**
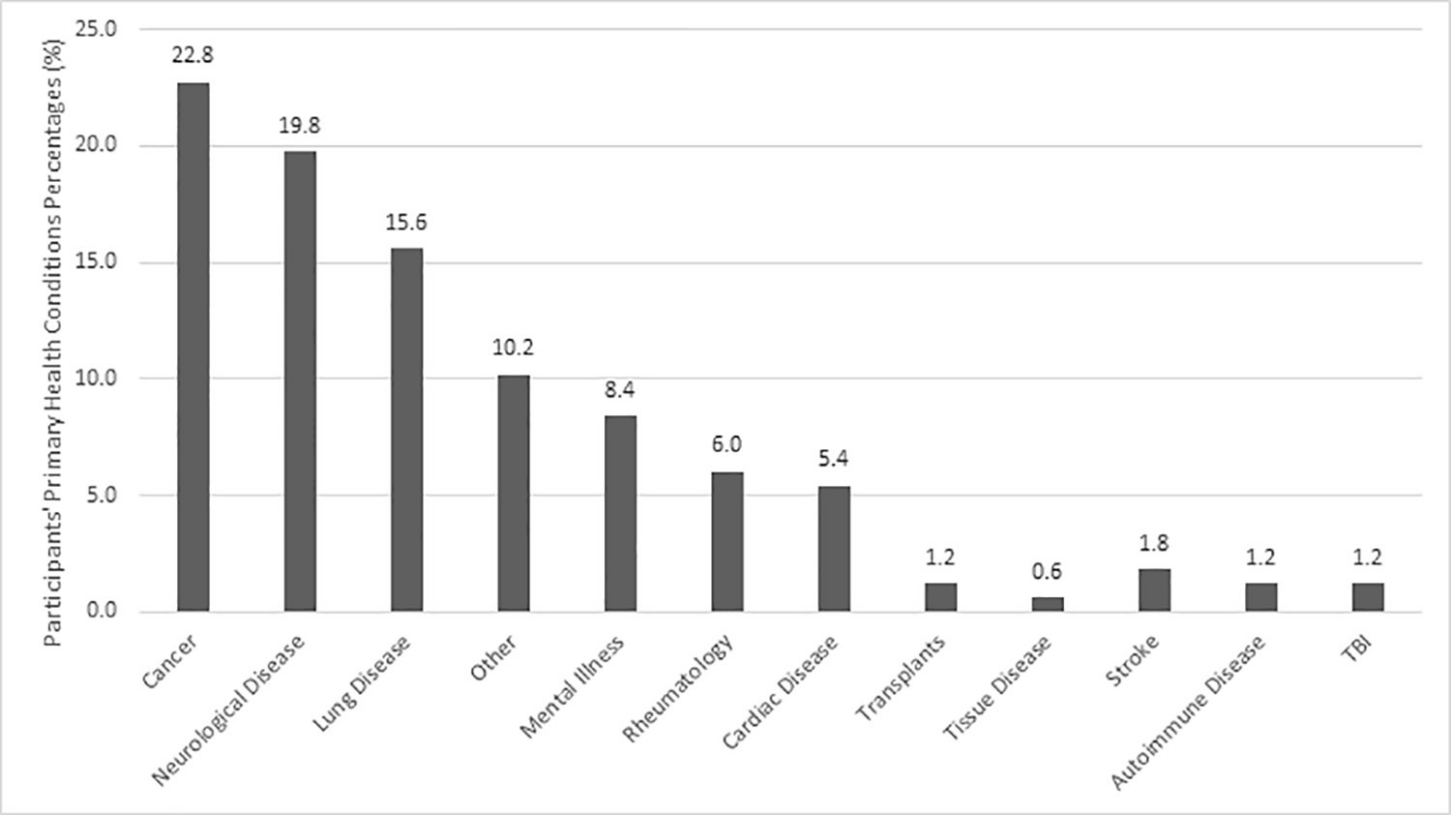
Participants’ primary health condition percentages (grouped by disease category).

Overall, sociodemographic and clinical characteristics were comparable across both study phases. Fig 1 shows the variety of primary conditions experienced by the study participants, of which the most common was cancer (23%), neurological conditions (20%), lung diseases (15%), mental illnesses (8%), and cardiac condition (5%). Patients resided throughout the United States, but most were from California (8%), Florida (7%), Texas (6%), Wisconsin (6%), Massachusetts (5%), and Minnesota (5%).

### Psychometric assessment of the SCCCS

#### Classical test theory

Table 2 shows the main psychometric analyses of the SCCCS using Classical Test Theory.

**Table 2:** Psychometric properties of the SCCCS using classical test theory.

	Mean (SD), n (%), or correlation coefficient		Floor Effect, n (%)	Ceiling Effect, n (%)	Item-to-total correlation (EFA)
Cronbach’s Alpha	—		—	—	0.98
SCCCS					
1. Express sensitivity, caring and compassion for your situation	7.6 (2.6)		7 (4.2)	54 (32.3)	0.89
2. Strive to understand your emotional needs	6.8 (2.8)		11 (6.6)	34 (20.4)	0.89
3. Consider the effect of your illness on you, your family and the people most important to you	6.6 (2.9)		16 (9.6)	37 (22.2)	0.83
4. Listen attentively to you	7.4 (2.7)		10 (6.0)	50 (30.0)	0.92
5. Convey information to you in a way that is understandable	8.0 (2.5)		7 (4.2)	71 (42.5)	0.86
6. Gain your trust	7.6 (2.9)		10 (6.0)	63 (37.7)	0.93
7. Always involve you in decisions about your treatment	7.5 (2.8)		12 (7.2)	55 (32.9)	0.87
8. Comfortably discuss sensitive, emotional or psychological issues	6.6 (3.1)		18 (10.8)	38 (22.8)	0.91
9. Treat you as a person not a disease	7.8 (2.7)		8 (4.8)	70 (41.9)	0.93
10. Show respect for you, your family and those important to you?	7.9 (2.8)		9 (5.4)	67 (40.1)	0.91
11. Communicate results in a timely and sensitive manner	7.3 (2.9)		12 (7.2)	48 (28.7)	0.87
12. Spend enough time with you	6.8 (3.1)		17 (10.2)	42 (25.2)	0.91
Total Score	87.9 (30.1)		2 (1.3)	11 (6.9)	-
CARE scores					
1. Making you feel at ease	Poor	11 (6.6)			
	Fair	19 (11.5)			
	Good	22 (13.3)			
	Very Good	58 (35.0)			
	Excellent	56 (33.7)			
	NA	1			
2. Letting you tell “your story”	Poor	13 (7.8)			
	Fair	23 (13.8)			
	Good	30 (18.0)			
	Very Good	51 (30.5)			
	Excellent	50 (30.0)			
	NA	0			
3. Really listening	Poor	16 (9.6)			
	Fair	23 (13.9)			
	Good	35 (21.1)			
	Very Good	42 (25.3)			
	Excellent	50 (30.1)			
	NA	1			
4. Being interested in you as a whole person	Poor	19 (11.5)			
	Fair	22 (13.3)			
	Good	32 (19.3)			
	Very Good	40 (24.1)			
	Excellent	53 (31.9)			
	NA	1			
5. Fully understanding your concerns	Poor	19 (11.5)			
	Fair	24 (15.6)			
	Good	31 (18.8)			
	Very Good	49 (29.7)			
	Excellent	42 (25.5)			
	NA	2			
6. Showing care and compassion	Poor	15 (9.2)			
	Fair	22 (13.4)			
	Good	30 (18.3)			
	Very Good	39 (23.8)			
	Excellent	58 (35.4)			
	NA	3			
7. Being positive	Poor	10 (6.1)			
	Fair	16 (9.8)			
	Good	35 (21.3)			
	Very Good	52 (31.7)			
	Excellent	51 (31.1)			
	NA	3			
8. Explaining things clearly	Poor	8 (4.8)			
	Fair	21 (12.7)			
	Good	27 (16.3)			
	Very Good	57 (34.3)			
	Excellent	53 (21.9)			
	NA	1			
9. Helping you take control	Poor	13 (8.0)			
	Fair	21 (3.0)			
	Good	34 (21.0)			
	Very Good	51 (31.5)			
	Excellent	43 (26.5)			
	NA	5			
10. Making plan of action with you	Poor	17 (10.4)			
	Fair	23 (14.0)			
	Good	32 (19.5)			
	Very Good	40 (24.4)			
	Excellent	52 (31.7)			
	NA	3			
Total Score CARE	36.3 (11.7)				
Psychometric Properties					
Convergent Validity (Pearson’s ρ)	0.77 (p<0.0001)				
Test-Retest Reliability (ICC 1,2, 95% CI)	0.90 (0.82–0.94)				

SD: Standard Deviation; EFA: Exploratory factor analysis.

SCCCS: Theoretical and actual ranges for all items: 1–10, for total score: 12–120; CARE: Theoretical and actual ranges for all items: 1–5; for total score: 10–50

The presence of potential floor and ceiling effects were first observed. Floor effects were not present for any of the items of the SCCCS. A ceiling effect could however be present for some questions such as *“3*: *Consider the effect of your illness on you*, *your family and the people most important to you”* (42.5%), *“9*: *Treat you as a person not a disease”* (41.9%) or *“10*: *Show respect for you*, *your family and those important to you”* (40.1%). Items with ceiling effect are typically further examined if there are problems with the other psychometric properties of the measure. We further explored the results of the CTT findings with RMT with particular focus on these three items.

Internal consistency reliability was high, with an overall Cronbach’s alpha coefficient of 0.98, indicating excellent internal consistency and far beyond the minimum 0.7 level recommended [22]. Table 2 also shows that item-to-total correlations were excellent, ranging from 0.83 to 0.93. Convergent validity (construct) was confirmed by a moderate and positive correlation of 0.77 (p<0.0001) between the SCCCS and the CARE. Test-retest reliability was assessed by comparing the consistency of scoring of the SCCCS at the two administration times using Intraclass Correlation Coefficients (ICC 1,2), and was also excellent, with a test-retest reliability of 0.90 (95% Confidence Interval: 0.82–0.94).

An exploratory factor analysis was performed, and a one-factor solution was deemed to be most optimal. Factorial validity was assessed using Confirmatory Factor Analysis, the Root Mean Square Residual was 0.03 (0.08 or less for acceptable model fit and the Bentler Comparative Fit Index was 0.92 (0.9 or more for acceptable model fit), which is indicative of good fit [26,27,28]. A Chi-square probability of < 0.0001 and the Root Mean Square Error of Approximation of 0.15 did not support acceptable model fit. Other goodness of fit indices were not reported due to the current consensus of not using these measures due to lack of stability [29]. Due to the conflicting fit statistics, RMT results were examined before reaching conclusions on the dimensionality of the measure.

#### Rasch measurement theory

We further evaluated the scaling properties and construct validity of the SCCCS using RMT. Table 3 summarizes the RMT analyses. Fit to the RMT model was examined using fit residual mean values between the expected scores and the actual score. A perfect fit would be indicated by a summary mean of zero and standard deviation of ±1: our item residual was of 0.13 ± 1.13 and person residual of -0.53 ± 1.76, both being examined for fit to a model in RMT The Chi-square probability of the item to trait interaction estimated the invariance of the scale (indicative of a significant deviation and a probability value of greater than 0.05 was not achieved (p = 0.04), which supports model fit. The PSI, an analogous coefficient to Cronbach’s alpha (and similar values to indicate good reliability), was 0.97, indicating high reliability.

**Table 3:** Summary rasch fit statistics and psychometric criteria of the SCCCS.

Analysis	Item Residual	Person Residual	Chi-Square		Reliability (PSI)	Unidimen-sionality * t test (95% CI)	% of t tests significant
	Mean (SD)	Mean (SD)	χ^2^ (df)	*p*			
Initial SCCCS	0.13 (1.13)	-0.53 (1.76)	37.3 (24)	0.04	0.97	0.03 (-0.003–0.06)	8.6%
SCCCS rescored	0.08 (1.06)	-0.56 (1.78)	34.0 (24)	0.09	0.97	0.03 (-0.003–0.06)	8.6%

SD: Standard deviation; χ^2^: Chi-square, df: degrees of freedom, PSI: Person Separation Index, CI: Confidence Interval

* No response dependency was observed.

**Fig 2:**
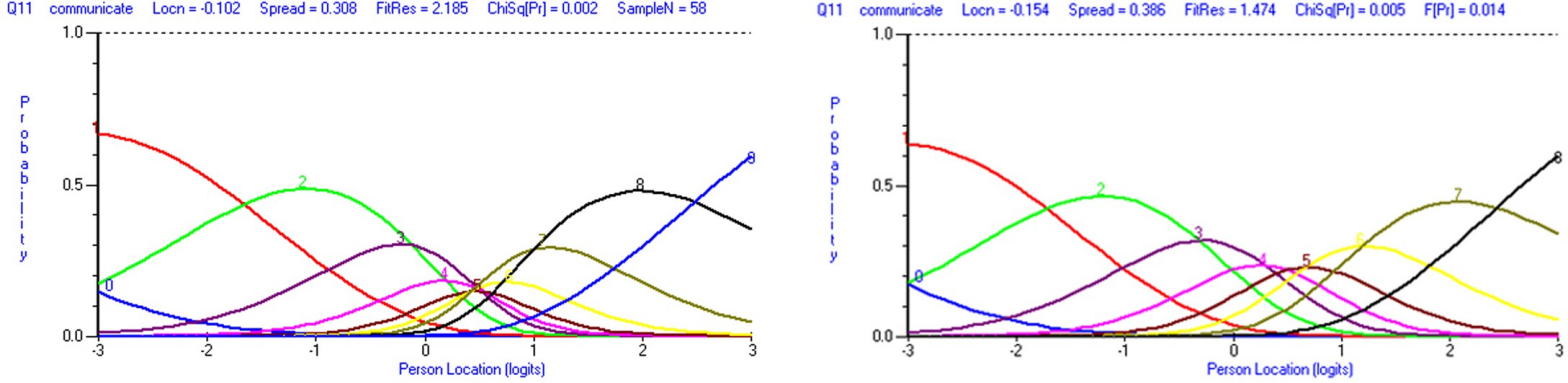
Category probability curves for item 11 (communicate results in a timely and sensitive manner) before and after rescoring response options. In panel 2a, category probability curves for item 11 show disordered thresholds. The x-axis is the construct, with the degree of compassionate care increasing from left to right. The y-axis shows the probability of endorsing the response categories 0 to 9 (10 response options in total). In panel 2b, category probability curves for item 11 show ordered thresholds after collapsing two adjacent response options together. The x-axis is the construct, with the degree of compassionate care increasing from left to right. The y-axis shows the probability of endorsing the response categories 0 to 8 (9 response options in total).

Fig 2 illustrates the item category thresholds results. If the response options follow a logical order, the transition points between a response category and another should be ordered from low to high. Fig 2A, for instance, illustrates item 11 which measures whether the physician communicates results in a timely and sensitive manner. Response options ranged from *Not at all Successful* (1) to *Very Successful* (10). Fig 2A shows that the transition points (or thresholds) between 3 and 4, 4 and 5, 5 and 6, and 6 and 7 were disordered, resulting in little discrimination between two response options for item 11. We collapsed categories of all items with disordered thresholds (10, 11, and 12) to improve the fit of the measure to the RMT model. Fig 2B illustrates how rescoring the response options to 9 improved the order of threshold for item 11.

Table 3 (SCCCS rescored) shows the improvement in model fit after three items were minimally rescored from 10 to 7 (item 10) or to 9 (items 11 and 12) response options. Item residual and person residual were improved, and the Chi-square probability obtained was acceptable (0.09, so above the statistically significance level of 0.05). Internal reliability remained high at 0.97.

Local dependency was evaluated using unidimensionality and response dependency. The unidimensionality of the SCCCS was assessed using independent t-tests between subsets of items identified by a principal component analysis of the residuals. Table 3 shows that 8.6% of *t*-tests were statistically significant (above the recommended level of 5%). The mean *t*-test value and 95% CI (0.03 (-0.003–0.06)) support acceptable unidimensionality of the SCCCS. The magnitude of residuals assessed the degree of response dependency, a situation where two items’ answers are so correlated that it could overestimate reliability indices. It confirmed the absence of response dependency.

**Fig 3:**
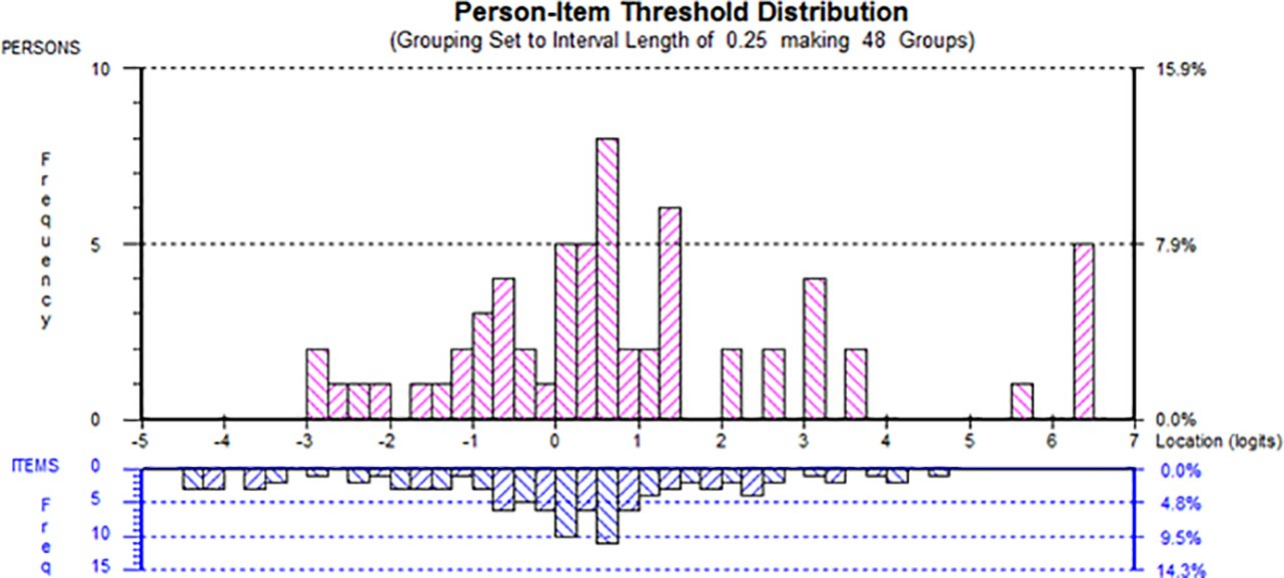
Person-item threshold distribution of the SCCCS. Person-Item thresholds. The items are shown at the reversed histogram at the bottom and the patient samples is shown in the top histogram. The figure shows that targeting between person (top) and the items (bottom). There is a lack of coverage of items at the highest degree of compassionate care.

Targeting was estimated by the match of patients’ ratings of compassionate care and the level of compassionate care measured by the items and response options. Fig 3 shows the person-item threshold distribution of the SCCCS. The top histogram shows the estimated evaluations of compassionate care by patients, ranging from low (left of the scale) to high (right of the scale). On the same scale, the item thresholds levels, range from low levels of compassionate care (left of the scale) to high levels (right of the scale). A first observation is that there are higher levels of compassionate care assessments of physicians than what the scale permits; that is, patients are rating compassionate care of physicians lower than their level of compassion simply because the items do not permit a higher evaluation. Similarly, there are items at the lower level of compassions where patients did not seem to encounter that level of uncompassionate care.

Finally, we examined whether groups of patients responded to questions differently either by consistently answering the same responses (Uniform DIF) or inconsistently (Non-Uniform DIF). Uniform and Non-Uniform DIF was assessed for gender, age group, household income, ethnic group, and time since admission using analysis of variance (ANOVA, alpha = 0.05). None of the group classifications created DIF.

Table 4 demonstrates that all items (items 10, 11, and 12 being rescored) matched the RMT (Chi-square p-value > 0.05). Moreover, the items are ordered from the lowest level of compassionate care (*5*. *Conveying information in a way that is understandable*) to highest level of compassionate care *(3*. *Considering the effect of your illness on you*, *the family*, *and people most important to you)* as determined by the RMT model. This information could be useful if one would wish to develop a brief SCCCS version or only administer a few items.

**Table 4:** Rasch measurement theory statistical indicators of item fit by location order.

Item	Rescored (y/n)	Item location	SE	Fit Residual	Chi-square	DF	*p*-value
*5*. *Convey information to you in a way that is understandable*	n	-0.69	0.12	1.49	1.88	2.00	0.39
*9*. *Treat you as a person not a disease*	n	-0.31	0.11	-1.21	2.15	2.00	0.34
*1*. *Express sensitivity*, *caring and compassion for your situation*	n	-0.30	0.12	-0.80	2.86	2.00	0.24
*10*. *Show respect for you*, *your family and those important to you*?	y	-0.29	0.12	0.10	1.24	2.00	0.54
*11*. *Communicate results in a timely and sensitive manner*	y	-0.15	0.11	1.47	10.63	2.00	0.00
*6*. *Gain your trust*	n	-0.05	0.11	-0.29	1.24	2.00	0.54
*7*. *Always involve you in decisions about your treatment*	n	-0.02	0.10	1.49	4.30	2.00	0.12
*4*. *Listen attentively to you*	n	-0.02	0.11	0.29	0.91	2.00	0.64
*2*. *Strive to understand your emotional needs*	n	0.28	0.11	-0.56	1.30	2.00	0.52
*12*. *Spend enough time with you*	y	0.50	0.12	-0.35	0.54	2.00	0.76
*8*. *Comfortably discuss sensitive*, *emotional or psychological issues*	n	0.51	0.11	-1.52	4.87	2.00	0.09
*3*. *Consider the effect of your illness on you*, *your family and the people most important to you*	n	0.54	0.11	0.86	2.04	2.00	0.36

SE: Standard Error; DF: Degree of Freedom

## Discussion and conclusions

CTT psychometric analyses showed that the 12-item SCCCS demonstrated excellent internal consistency reliability (α = 0.98), far beyond the minimum 0.7 level recommended [20], and also supported by high item-to-total correlations. Patients’ ratings of compassionate behaviors correlated statistically significantly with the CARE, a measure of empathy, a related construct, confirming convergent validity. The unidimensionality of the SCCCS was assessed with exploratory and confirmatory factor analysis, but not all fit statistics reported confirmed a one-factor solution. A potential reason is that the sample size for which a general recommendation may be made is 5 to 10 respondents per parameter [26]. With 24 parameters in our 1-factor model, a sample of 120 to 240 patients would be needed, and our 167 participants meet barely the minimal requirements to conduct the analyses. An additional limitation related to sample size is that for variables that have non-normal distribution, the Chi-square test (and Root Mean Square Error of Approximation which is based on the Chi-square test) is not a reasonable measure of fit [26]. An additional support for the 1-factor solution, however, is RMT, which confirmed the unidimensionality of the SCCCS. Common guidance for RMT recommend at least 150 respondents for results with 99% confidence [30]. Test-retest reliability was evaluated for the first time and was also determined to be excellent (r = 0.90 (0.82–0.94)), although with only 61 participants completing the retest section, this could also be seen as a sample size limitation. Further research with larger sample sizes may result in additional findings.

RMT psychometric analyses were used to evaluate the scaling properties and construct validity of the SCCCS. The first improvement of the measure was done by rescoring three items of the SCCCS (items 10, 11, and 12). Previous research has supported that the optimal response options for surveys should not go above 7 categories due to the cognitive load of managing and making decisions about managing the presented information [31]. RMT analyses demonstrate that rescoring a few items to 7 to 9 response options improves the fit to the RMT model. The psychometric properties of the measure are improved as a result, but for further improvement of the measure it would be recommended to cognitively debrief patients again on whether a 10 point Likert-scale is appropriate for this measure, comparing it with 7 to 9 response options, and reassessing the fit of the model with all items rescored to 7, 8, and 9 response options, as homogeneous response options throughout a scale is often preferred.

With the improvement in fit following the rescoring of 3 items, most of the RMT analyses demonstrated excellent psychometric properties: no items were associated with DIF, the fit statistics were good to excellent, and the reliability was high. Local dependency was evaluated using response dependency and unidimensionality, and the measure was confirmed to be unidimensional. This was also the case of the 16-item version of the SCCCS [14].

Removing or adding items to the 12-item version of the SCCCS could potentially address the person-item threshold distribution which demonstrated that patients may wish to rate compassionate care at levels both higher and lower than the SCCCS permits. Nevertheless, we were able to order the current 12 items of the SCCCS on an interval scale from low to high levels of compassionate care, a first basis for the application of a computerized adapted testing design for the administration of the SCCCS.

Although compassionate care is not routinely assessed, compassion is associated with outcomes that are important to patients, family members and healthcare professionals, and these groups endorse its importance. The SCCC has undergone extensive qualitative and quantitative assessments leading to a robust 12-item version. Compared with existing measures, the SCCCS adds items related to understanding and discussing emotional and contextual issues, and the needs of the patient as well as those he or she considers family.

This study does have some limitations. Although the SCCCS has been improved and is demonstrated to be a valid and reliable measure, there was a selection bias. The PatientsLikeMe patient community, as most health-related online patient samples, have been shown to be slightly younger, more actively engaged in their health, and more educated than the general population, generally more representative of a White ethnic background, and more female. An additional factor adding to the selection bias is the low number of respondents completing the questionnaire in comparison to those who were invited to participate, which is typical of online surveys by email invitation. Additional studies are needed in other settings and with more varied populations, including with Hispanic patients and in languages other than English. Moreover, we included patients hospitalized within the last 18 months, but a potential recall bias may be present among such patients. Repeating the study with a shorter time frame may confirm whether recall bias is present in the current sample.

A final limitation to the interpretation of these results is based in the theoretical underpinnings of the concept being measured, compassionate care. A noted observation was that certain items were skewed in favor of a high ceiling effect. It would be of interest to compare results across samples recruited from institutions with high publicly available patient experience or satisfaction ratings as compared with those institutions with lower ratings to see if these ceiling effects noted are similarly affected.

### Practice implications

We recognize that many healthcare professionals are finding the current measurement environment and requirements burdensome. Nevertheless, individuals and organizations who wish to improve patients’ perceptions of the quality of their care and communication should be encouraged to use valid and reliable instruments to uncover gaps between what patients and professionals believe is important and what professionals are able to provide. Of note in this study is the fact that three of the lowest ratings by non-hospitalized patients with multiple chronic conditions were those related to considering the effects of illness on patients and their families; to discuss sensitive, emotional, or psychological issues; and to spend enough time with the patient is the essence of patient-centered care. Some of these components are reflected in patient surveys that impact federal payments to hospitals in the U.S. and thus have significant financial implications [32]. The SCCCS could be used to provide more specific, tailored feedback to individual professionals. It could also be used as a patient-reported outcome measure to assess the impact of organizational interventions and initiatives on individual-, unit-, team- and clinic-level capacity to provide compassionate care. Further testing of correlations among a smaller group of items or of a global item with the SCCCS is also warranted. If found to be valid and reliable, we recommend that such an item or items be included in nationally used patient experience and satisfaction surveys.

## Supporting information

S1 Table The Schwartz Center Compassionate Care Scale. (DOCX)

S2 Table Cognitive debriefing methods, participant characteristics and results. (DOCX)

S3 Table Wording, syntax and semantics of the SCCCS. (DOCX)
