# Correction: Measuring compassionate healthcare with the 12-item Schwartz Center Compassionate Care Scale

**DOI:** 10.1371/journal.pone.0223852

**Published:** 2019-10-16

**Authors:** Ana Maria Rodriguez, Beth A. Lown

## Notice of republication

Incorrect versions of S1–S3 Tables were published in error. This article was republished on September 27, 2019 to correct for these errors. Please download this article again to view the correct version.
