# Supplementary material for: Measuring compassionate healthcare with the 12-item Schwartz Center Compassionate Care Scale
Source: PLoS One. 2019 Sep 5;14(9):e0220911. doi: 10.1371/journal.pone.0220911 (PMC6728044; doi:10.1371/journal.pone.0220911)
Supplement: S1 Table — (DOCX) [file pone.0220911.s001.docx]

**S1 Appendix: The Schwartz Center Compassionate Care Scale®**

**Schwartz Center Compassionate Care Scale (Patient Version)®**

Compassionate care involves relating well to patients and families and recognizing and addressing their concerns and distress. Healthcare professionals’ compassionate care is essential to high quality medical care.

Please answer the following questions with regard to the doctor who was in charge of your care during your recent hospitalization.

**On a scale of 1 to 10, where 1 is not at all successfully and 10 is very successfully, how successfully did your doctor:**

|  |  | 1 | 2 | 3 | 4 | 5 | 6 | 7 | 8 | 9 | 10 |
| --- | --- | --- | --- | --- | --- | --- | --- | --- | --- | --- | --- |
| 1 | Express sensitivity, caring and compassion for your situation | 🔾 | 🔾 | 🔾 | 🔾 | 🔾 | 🔾 | 🔾 | 🔾 | 🔾 | 🔾 |
| 2 | Strive to understand your emotional needs | 🔾 | 🔾 | 🔾 | 🔾 | 🔾 | 🔾 | 🔾 | 🔾 | 🔾 | 🔾 |
| 3 | Consider the effect of your illnesses on you and your family | 🔾 | 🔾 | 🔾 | 🔾 | 🔾 | 🔾 | 🔾 | 🔾 | 🔾 | 🔾 |
| 4 | Listen attentively to you | 🔾 | 🔾 | 🔾 | 🔾 | 🔾 | 🔾 | 🔾 | 🔾 | 🔾 | 🔾 |
| 5 | Convey information in a way that is understandable | 🔾 | 🔾 | 🔾 | 🔾 | 🔾 | 🔾 | 🔾 | 🔾 | 🔾 | 🔾 |
| 6 | Gain your trust | 🔾 | 🔾 | 🔾 | 🔾 | 🔾 | 🔾 | 🔾 | 🔾 | 🔾 | 🔾 |
| 7 | Involve you in decisions about your treatment | 🔾 | 🔾 | 🔾 | 🔾 | 🔾 | 🔾 | 🔾 | 🔾 | 🔾 | 🔾 |
| 8 | Comfortably discuss sensitive, emotional or psychological issues | 🔾 | 🔾 | 🔾 | 🔾 | 🔾 | 🔾 | 🔾 | 🔾 | 🔾 | 🔾 |
| 9 | Treat you as a person not just as a disease | 🔾 | 🔾 | 🔾 | 🔾 | 🔾 | 🔾 | 🔾 | 🔾 | 🔾 | 🔾 |
| 10 | Show respect for you and your family | 🔾 | 🔾 | 🔾 | 🔾 | 🔾 | 🔾 | 🔾 | 🔾 | 🔾 | 🔾 |
| 11 | Communicate test results in a timely and sensitive manner | 🔾 | 🔾 | 🔾 | 🔾 | 🔾 | 🔾 | 🔾 | 🔾 | 🔾 | 🔾 |
| 12 | Spend enough time with you | 🔾 | 🔾 | 🔾 | 🔾 | 🔾 | 🔾 | 🔾 | 🔾 | 🔾 | 🔾 |
