# Supplementary material for: Measuring compassionate healthcare with the 12-item Schwartz Center Compassionate Care Scale
Source: PLoS One. 2019 Sep 5;14(9):e0220911. doi: 10.1371/journal.pone.0220911 (PMC6728044; doi:10.1371/journal.pone.0220911)
Supplement: S2 Table — (DOCX) [file pone.0220911.s002.docx]

**S2. Cognitive Debriefing Methods, Participant Characteristics and Results**

**Methods:**

PLM patient-members were cognitively debriefed electronically about the content, the wording and the meaning of the 12 items of the SCCCS and the response options used. Participants were asked to evaluate each item and response option by indicating whether the question applied to them, how easy the question was to understand, and how well the response choices fit their understanding of each question.

In addition, the wording, syntax, and semantics of the SCCCS items were examined using the “Question Understanding Aid,” a system that uses a variety of computational linguistics techniques to locate potential problems in questionnaire items. A list of potential problems for each item was generated, including (1) unfamiliar technical term, (2) vague or imprecise relative term, (3) vague or ambiguous noun-phrase, (4) complex syntax, (5) working memory overload, and (6) misleading or incorrect presupposition.

**Sociodemographic Characteristics of the Cognitive Debriefing Participants:**

| *Characteristics* | **Mean (SD) or Frequency (%)**  **Total n =23** |
| --- | --- |
| Age | 57.7 (13.1)  (Range: 33-75) |
| Age Group |  |
| 18 to 39 years | 3 (13.0) |
| 40 to 55 years | 5 (21.7) |
| 56 to 65 years | 7 (30.4) |
| Above 65 years | 8 (34.8) |
|  |  |
| Gender |  |
| Female | 8 (65.2) |
| Male | 15 (34.8) |
|  |  |
| Ethnicity |  |
| Non-Hispanic | 22 (95.7) |
|  |  |
| Education (missing n=4) |  |
| Some high school or High School | 3 (13.0) |
| Some College | 12 (52.2) |
| College | 4 (17.4) |
| Graduate Education | 4 (17.4) |
|  |  |
| Household Income (missing n=26) |  |
| 0-25K$ | 7 (30.4) |
| 36-50K$ | 5 (21.7) |
| 50-100K$ | 3 (13.0) |
| 100-200K$ | 2 (8.7) |
| 200K$ and above | 1 (4.4) |
|  |  |
| Time Since Last Hospital Admission |  |
| Within the last 90 days | 2 (8.7) |
| Within the last 6 months | 1 (4.4) |
| Within the last 12 months | 14 (60.9) |

**Results:**

The items of the SCCCS were assessed in terms of whether they were easy to understand, whether they applied well to the patient’s situation, and whether the response choices how well the response choices fit their understanding of each question. All patients surveyed indicated that the questions were “very easy” or “reasonably easy” to understand, that they applied “reasonably well” or “very well” to their situation, and that the response options fit the questions “reasonably well” or “very well.” Ranges varied across items, but no one indicated an unfit understanding, application, or response option during the cognitive debriefing survey.

The interest however lies in the suggestions given by patients to improve the SCCCS. A few patients mentioned including the possibility of not rating a physician on an item:

“*My doctor didn't address emotional needs at all. If he had tried, he may have been very successful. I think that there needs to be an answer that reflects this option because an "unsuccessful" rating eludes to him doing a terrible job.”*

*“Give a "does not apply" answer or something equal to that for each question.”*

A possible source of confusion for patients was identified as to whom the measure was assessing, as a patient may have different conditions or even for a single condition, might have to relate with several physicians:

*“State explicitly who we are rating...the clinician in charge of our last hospitalization or the clinician we normally see to treat our illness. It is often different people, as it was in my case.”*

*“Maybe not specific enough, specify for which doctor and not only after admission to a hospital”*

*“I had two surgeries within days of each. Therefore, two different surgeons handling my care. One was incredibly compassionate the other, not so compassionate.”*

*“Most of us have more than one condition. I think it would be helpful to somehow acknowledge this in the questionnaire.”*

The syntactic analysis of the questions of the SCCCS also indicate that additional precisions could be provided regarding the use of certain words. As seen in Appendix 3, terms such as “sensitivity”, “caring”, “compassion”, “attentively”, “understandable”, “comfortably”, and “timely” could be unfamiliar to some respondents. Identified vague or ambiguous nouns were the use of the terms “needs”, “illness”, and “family”. Finally, the terms “most” and “always” were determined to be possible sources of frequency ambiguity (a respondent is asked to rate a precise point about a prolonged behavior).

The cognitive debriefing survey was brief but provided valuable insights to potentially improve some aspects of the measure. Patients articulated wanting to identify whether an item did not apply to their physician so as to not rate them unfairly. Patients also wanted more precision regarding the physician they were rating, as they relate with several physicians and often have co-morbidities. Identifying the physician and/or primary condition could meet this preference.

The syntactic and semantic analysis of the SCCCS was useful in providing guidance for minor improvement in the words used that might not be understood similarly by all patients. Including adverbs such as always, never, or most should be avoided as the item could be quantified differently, which would affect the validity of the item. Besides these suggestions for improvement however, the 12-item SCCCS version seem to have good to excellent internal validity.
