# Supplementary material for: Measuring compassionate healthcare with the 12-item Schwartz Center Compassionate Care Scale
Source: PLoS One. 2019 Sep 5;14(9):e0220911. doi: 10.1371/journal.pone.0220911 (PMC6728044; doi:10.1371/journal.pone.0220911)
Supplement: S3 Table — (DOCX) [file pone.0220911.s003.docx]

**S3. Wording, Syntax, and Semantics of the SCCCS items using the Question Understanding Aid**

| **SCCCS Item** | **Unfamiliar technical terms** | **Vague or imprecise relative terms** | **Vague or ambiguous noun-phrase** | **Complex Syntax** | **Working memory overload** |
| --- | --- | --- | --- | --- | --- |
|  | *The following term may be unfamiliar to some respondent* | *The following terms refer implicitly to an underlying continuum or scale, but the point or value on the scale is vague or imprecise* | *The referent of the following nouns or pronouns may be vague or ambiguous to the respondent* | *Question or Answer is either ungrammatical or difficult to parse syntactically* | *Question or answer imposes a heavy load on the working memory of the respondent* |
| 1. Express sensitivity, caring and compassion for your situation | Sensitivity, Caring, Compassion |  |  |  |  |
| 2. Strive to understand your emotional needs |  |  | Needs |  |  |
| 3. Consider the effect of your illness on you, your family and the people most important to you |  | Frequency ambiguity: Most | Illness, Family |  |  |
| 4. Listen attentively to you | Attentively |  |  |  |  |
| 5. Convey information to you in a way that is understandable | Understandable |  |  |  |  |
| 6. Gain your trust |  |  |  |  |  |
| 7. Always involve you in decisions about your treatment |  | Frequency ambiguity: Always |  |  |  |
| 8. Comfortably discuss sensitive, emotional or psychological issues | Comfortably |  |  |  |  |
| 9. Treat you as a person not a disease |  |  |  |  |  |
| 10. Show respect for you, your family and those important to you? |  |  | Family |  |  |
| 11. Communicate results in a timely and sensitive manner | Timely |  |  |  |  |
| 12. Spend enough time with you |  |  |  |  |  |
